# Dialogic Social Impact Analysis of Companies and Organizations (DSIACO): A pioneer model for evaluating social impact of companies and organizations

**DOI:** 10.1371/journal.pone.0334833

**Published:** 2025-10-27

**Authors:** Mar Joanpere, Ana Burgues-Freitas, Cristina Pulido, Adriana Aubert, Elisabeth Torras-Gómez, Maria Vieites, Marta Soler-Gallart, Ramon Flecha

**Affiliations:** 1 Department of Business and Management, Universitat Rovira i Virgili, Tarragona, Spain; 2 Department of Sociology, University of Granada, Granada, Spain; 3 Department of Journalism and Communication Sciences, Universitat Autònoma de Barcelona, Barcelona, Spain; 4 Department of Sociology, University of Barcelona, Barcelona, Spain; 5 University of Barcelona, Barcelona, Spain; Universiti Teknologi MARA, MALAYSIA

## Abstract

Scientific literature has clarified that companies’ social impact is crucial in improving society by optimising their performance and relationship with their stakeholders, creating long-term value. Companies are increasingly interested in assessing their social impact. For instance, applying regulatory compliance increases investors’ confidence, reputation, and image. It reduces risks and costs and improves decision-making, resilience, and talent retention. The concept of social impact and the scientific basis for evaluating and enhancing it have also been clarified and officially published by international organisations (besides the scientific literature). However, there is a lack of scientific literature on how to analyse companies’ social impact based on scientific evidence of social impact. Building upon the latest contributions of the ALLINTERACT research project, funded by the European scientific programme Horizon 2020, we have focused on analysing the social impact of companies and institutions. Our work is based on the communicative methodology of research, which pioneered the development and proposal of the criteria of social impact and co-creation. In this regard, a twofold goal is addressed in this study. On the one hand, a systematic literature review has been conducted to identify existing models of social impact evaluation for companies and organisations based on scientific evidence of social impact. On the other hand, the Dialogic Social Impact Analysis of Companies and Organisations model for assessing social impact tailored for companies and institutions is presented, which has been previously developed as a result of scientific research about social impact evaluation in different fields. The results of the systematic literature review extracted from the screening of 387 articles and analysis of 70 of those articles revealed the absence of any model developed enough to assess companies’ social impact based on scientific evidence of social impact. Existing models make critical errors like confusing social impact with transference. Hence, the DSIACO model presented in this article addresses this critical gap and drives scientific advancements in this area.

## Introduction

Scientific evidence of social impact plays a crucial role in addressing societal needs. Social impact refers to the social improvements that advance towards the achievement of society’s goals. The concept of social impact was introduced in science as a response to citizens’ and various institutions’ demands for scientific research to demonstrate its actual or potential social impact, that is, the improvements advancing towards societal goals as a result of the evidence and knowledge generated through research [1]. To achieve and promote the social impact of research, there is a need for co-creation, which involves engaging diverse citizens in ongoing, egalitarian dialogue. Co-creation happens throughout the entire scientific endeavour, from the design of the research project to the assessment (also referred to as evaluation or measurement in this manuscript) of its achieved or potential social impact. It ensures that scientific knowledge is developed in collaboration with a diverse range of stakeholders, including individuals, governments, organisations, and businesses, and that it is the stakeholders themselves who assess the social impact of such knowledge [1]. Recognising this, major research programmes across all scientific disciplines are integrating the criteria of social impact and co-creation as requirements. These criteria have become central in guiding not only scientific inquiry but also the relationship between researchers, policymakers, and the public. The European Commission (EC) has taken the lead in embedding these principles within its scientific research framework, setting new standards for evaluating and improving social impact and co-creation outcomes. The EC enlisted top experts from all sciences, chaired by Flecha, who defined scientific evidence of social impact (SESI, acronym hereinafter) [2], to establish guidelines that help assess the social impact of research.

Whereas the social impact was created from science, it has been spread to the whole society and, as part of it, to companies and organisations. Indeed, by 2025, all large companies (those with more than 500 workers) in the European Commission will be required to regularly disclose reports of the social impact of their activities [3]. Social impact, therefore, refers to the actual or potential improvements advancing towards society’s goals as a result of company interventions; in other words, it relates to the value that the company brings to society. In this case, co-creation involves the participation of stakeholders in evaluating the social impact and in developing the product, service, or organisation to enhance its social impact. Stakeholder involvement is a broad concept that encompasses co-creation, as well as different types of involvement that do not involve co-creation.

Society is becoming more insistent in holding public and private institutions accountable for their contributions to social impact. Well-defined mechanisms are therefore needed to identify and measure the social advancements promoted by those companies and organisations. The academic community has been echoing this request for some time. Citizens want to be informed about the specific social improvements being pursued, which calls for higher transparency from businesses and resource providers. In line with this, social science researchers have been working for years to develop specific indicators to measure the social impact of their investigations accurately. In the current Dialogic Society [4], scientists’ task is not to decide society’s objectives but to provide citizens and their representatives with the analysis of the actions that contribute to achieving the objectives they have opted. These objectives are not merely a by-product of corporate growth but should align with socially agreed objectives, such as the Sustainable Development Goals (SDGs) developed by the United Nations (i.e., better health, well-being, or job security, among others) [5]. Social impact results from a company’s targeted interventions, policies, or services. However, it is important to note that current sustainability reports often focus more on disseminating and communicating social impact efforts rather than measuring their actual influence on people’s lives.

Pioneering studies employ a communicative methodology to assess social impact, including co-creation —a collaborative endeavour involving scientists, citizens, and organisations that focus on integrating diverse voices. This contribution achieves profound knowledge and social impact, avoiding stereotypes and fostering equitable dialogue [6, 7]. To accurately measure social impact, it is essential to have a clearly defined methodology grounded in scientific evidence of social impact [2]. This type of evidence is defined as follows “Scientific evidence of social impact (SESI) is scientifically backed evidence that, when taken as a basis for policies or actions, has led to improvements in society in relation to objectives that are the result of a broad consensus, which has resulted in decisions taken directly by citizens and/or through their representatives” [2, p.1]. This methodology should be subject to rigorous validation by the scientific community. The measurement of social impact should be multidimensional, considering the improvement of the lives of the company’s internal team, the target population of the corporation’s social responsibility initiatives, and individuals directly affected by the company’s daily operations.

In addition, interactive social impact [8] should also be considered. Interactive social impact refers to the improvements experienced not only by those directly targeted by a company’s interventions but also by individuals who interact with them, creating a ripple effect throughout the community. The relationship between social impact and interactive social impact can be understood as a layered or cascading effect, where the direct benefits experienced by the target population (social impact) create indirect benefits for those who interact with these initial beneficiaries (interactive social impact). Whereas social impact refers to the improvements experienced by individuals or communities directly targeted by a company’s intervention or research, interactive social impact extends this concept by capturing the indirect ripple effects on those who are not the primary targets but are influenced through their interaction with the primary beneficiaries. For example, suppose a company’s intervention improves the education of a community’s children (the target population). In that case, the parents, educators, and broader community who interact with these children might also experience benefits (interactive social impact), such as enhanced community cohesion or a better-educated future workforce. However, measuring social impact poses challenges due to the lack of consensus on specific metrics or indicators. On the one hand, systematically improving social impact measurement requires robust scientific evidence to ensure that the methods and metrics employed are valid, reliable, and subject to rigorous scrutiny. On the other hand, co-creation plays a critical role in enhancing the quality and inclusiveness of social impact assessments. Although not a formal requirement, co-creation enables the development of measurement tools that incorporate diverse perspectives and real-world experiences, thus strengthening the overall assessment process. By integrating both scientifically grounded evidence and the participatory element of co-creation, companies and organisations can enhance the rigour and relevance of their social impact measurements. This combination makes social impact assessment a more effective tool for contributing to societal transformation and aligning with increasing ESG (Environmental, Social, and Governance) regulations [9]. ESG refers to the standards used to assess the impacts of a company’s activities on the environment, society, and its corporate governance. In the last decades, ESG has been used as a means to assess whether to invest in a company or not [10]. In this regard, the Corporate Sustainability Reporting Directive (CSRD) defined three dimensions for evaluating and managing companies’ social impact among multiple stakeholder groups: 1) the internal team, referring to people who work for the company; 2) the target population, that is, people at whom the company’s social responsibility actions are aimed; and 3) the general population, meaning people affected by the company’s main daily activity [11].

Contrastingly, while many concur that organisations are experiencing a dialogical transformation [4] (that is, working more horizontally and democratically, engaging their members in egalitarian dialogues, and having social impact at the forefront), they deny this occurrence within corporations due to their primary aim of profit maximisation. Nevertheless, contemporary scientific examination reveals more dialogical undertakings in many businesses than ever. Rather than hindering their economic success, many are enhancing it through this dialogical transformation. It is not universal, though. The outcome hinges on the quality of their commitment to dialogue. Therefore, it is necessary for both the scientific community and society to closely examine businesses that are simultaneously advancing in both dialogue and economic success. Today’s businesses and academic institutions are actively working to address the urgent transformation towards a society that prioritises dialogue. This emphasis on dialogue serves as the cornerstone for democratisation and empowering citizens in articulating their needs for enhancing their lives [12].

While over the last years most corporations’ efforts have focused on highlighting their impact in the “E” (Environment) and “G” (Governance) without addressing the social aspect of sustainability, the inclusion of the “S” (Social aspect) in the ESG model [13] is not only urgent but also an essential step forward. We have entered an era where the ‘S’ in the ESG model has become more relevant than ever. This phenomenon can be attributed to the mounting societal demands for corporations to prioritise social responsibility alongside their business operations. This shift is significantly shaped by the United Nations’ Sustainable Development Goals (SDGs) framework. Although the SDGs provide a commendable blueprint for driving corporate accountability towards societal advancement, the escalating inequalities since their implementation demand a comprehensive re-evaluation. The UN’s future discussions in New York are anticipated to critically examine progress toward these goals and the collective societal responsibility, including that of corporations, in achieving them [9].

The development of dialogic models for assessing social impact, particularly those based on co-creation, addresses the limitations of traditional, monologic approaches by emphasising stakeholder involvement and mutual understanding. This model is especially vital for ensuring that social impact initiatives genuinely reflect the diverse values and needs of affected communities, as they are directly involved in defining, evaluating, and adapting the impact assessment process itself. Several sources highlight the importance of these dialogic approaches. Greene [14] emphasises that effective dialogic evaluation fosters inclusivity, respect, and equity, thus democratising the evaluation process and strengthening its legitimacy and accuracy. This approach requires a moral and ethical commitment to stakeholder engagement that respects diverse perspectives and seeks to balance power dynamics within the evaluative dialogue [14, 15]. Furthermore, it advocates for a stakeholder-based approach, arguing that such a framework helps mitigate manipulation risks and encourages genuine accountability. By involving multiple stakeholders in the creation and selection of social impact metrics, organisations can develop more accurate, contextually relevant assessments that avoid the pitfalls of one-size-fits-all standards. In addition, dialogic communication in social responsibility practices has shown tangible benefits in fostering trust, engagement, and sustained social impact. Studies in Corporate Social Responsibility (CSR, hereinafter) communication, such as those by Hurst et al. [16], demonstrate that dialogic, two-way communication helps organisations and stakeholders co-create shared values and empathy, which enhance both the authenticity and effectiveness of social impact initiatives [16]. Additionally, the relational approach to social impact aligns well with emerging frameworks in public relations and social enterprise, where fostering a fully functioning society relies on trust-based, reciprocal relationships between organisations and stakeholders [17, 18]. In summary, dialogic models of social impact assessment, rooted in co-creation, address the need for transparent, equitable, and meaningful stakeholder engagement.

In this sense, this study identifies a gap in the scientific literature on the assessment of companies’ social impact. Many companies are increasingly interested in assessing their social impact to achieve social impact as part of their business activities. However, whereas there is an increasing body of literature on assessing social impact in the research field, the evaluation of social impact based on SESI in the corporate field remains underexplored.

This study aims to address two objectives in order to fill this gap. The first aim is to conduct a state of the field on social impact evaluation in the corporate field to identify whether there is any model for assessing social impact in companies that collects SESI [2] and/or is based on co-creation, and what dimensions of the CSRD are considered in the studies analysed. The second aim is to present for the first time in a scientific manuscript the Dialogic Social Impact Analysis of Companies and Organisations (DSIACO) [19], which forges a new path in examining social impact by incorporating the most crucial scientific insights. DSIACO has been developed by the Community of Research on Excellence for All (CREA) since its inception in 1991, across various fields through scientific research [20]. As part of this endeavour, CREA has conducted several research studies on the social impact of different institutions [1, 20–30], developing the evaluation of social impact based on SESI in different fields. Whereas most of the scientific publications in this regard have focused on the evaluation of the social impact of institutions such as schools or universities [31–33], this is the first time that this theoretical contribution is presented in a scientific article. DSIACO develops analysis criteria previously unused in the context of companies, aiming to systematise the evaluation of their social impact and scientifically advance the understanding of social impact analysis and measurement. DSIACO’s approach aligns with the interests and objectives of multiple companies and provides a crucial framework for understanding and enhancing social impact within the corporate world. Based on Flecha’s Dialogic Society Theory [4] and the social impact evaluation based on SESI developed by Flecha and Soler [1, 24], DSIACO creates standard frameworks that can be applied in diverse contexts, facilitating a comprehensive understanding of a company’s social impact outcomes.

Having laid out the two main objectives of this study, the two research questions are as follows: R1: Is there any measurement of companies’ social impact based on scientific evidence of social impact and co-creation? And R2: What are the criteria of a model based on scientific evidence of social impact (SESI) for analysing the social impact of companies? To address R1, we have conducted a systematic literature review following the guidelines of the Preferred Reporting Items for Systematic Reviews and Meta-Analyses (PRISMA) [34] in Web of Science. To address R2, we have gathered and summarised evidence from previously performed scientific research (dating back to as early as 1991) on the social impact assessment, based on SESI and co-creation developed by CREA, as a dimension that improves social impact, although it is not a requirement. Through this twofold approach, this study aims to contribute an analysis designed to gauge the social impact of companies and organisations through a dialogical lens, specifically including the assessment of companies’ social impact based on SESI [2].

## Methods

To address both research questions set out in this study, we conducted two literature reviews. To address R1, we have decided to conduct a systematic literature review following PRISMA guidelines to obtain a transparent synthesis of the current state of knowledge [35] on existing assessments of companies’ social impact, identifying whether there are any based on SESI and co-creation. To address R2, we conducted a focused literature review of the scientific work undertaken by the Community of Research on Excellence for All (CREA), a research community that, since its founding in 1991, has dedicated its efforts to achieving social impact through evidence-based research in various fields. From this body of work, we present the Dialogic Social Impact Assessment of Companies (DSIACO) model, developed by CREA to analyse companies’ social impact through a dialogic approach grounded in SESI, therefore we present a model for analysing dialogically the social impact of companies based on SESI: DSIACO [19].

### Search strategy and eligibility criteria

Regarding the systematic literature review [36], the search strategy comprises several keyword combinations used as queries in Web of Science due to its high selectivity and rigorous indexing standards, which align with our study’s focus on capturing well-established, high-impact research within the current study. The selected period is from July 28, 2019, to July 28, 2023. The decision to focus on this specific period is based on an evaluation of the most recent literature review in this field. Table 1 defines the keywords used in each query.

**Table 1:** Keyword combinations used in queries conducted in web of science.

Keyword combinations	Query number
“social impact” AND companies	Query 01
“social impact” AND measurement AND companies	Query 02
“Environmental, Social, and Governance (ESG) measurement”	Query 03
“scientific evidence of social impact” AND companies	–
“scientifically validated evidence of improvement” AND companies	–

The source selected to conduct the search is Web of Science. The studies were selected based on their title, abstract, and relevance to the research questions, and the selection criteria were assessed. If a study met the inclusion criteria, the full-text article was retrieved for further analysis. The selection and appraisal of studies for inclusion also include in this review guided by the methodological contributions of [37], who developed the Quality Assessment Tool for Studies with Diverse Designs (QATSDD). In this case, the studies were selected based on their relevance to the following aspects: 1) Analysing how a company should assess its social impact, 2) Analysing the actual social impact of a company. Papers no focused on aspect 1 and 2 were excluded, duplicates nd those studies for which the full document could not be accessed were excluded.

### Screening and data extraction

We extracted the results from the Web of Science in Excel format. The obtained Excel sheet was adjusted to align with the specific information required by our research objectives. We retained the following details for each article: Authors, Article Title, Source Title, Author Keywords, Keywords Plus, Abstract, and DOI Link. Two additional columns were added. One of the added columns included the query number (see Table 1) through which each article was obtained. The other added column referred to the codes corresponding to the analysis categories, specifically whether the articles included SESI, co-creation, and/or any of the three dimensions outlined in the CSRD. For each of the analysed articles, a code was given for each analysis category: “0” was given if the article did not include the category, and “1” was given if it did include the category. Table 2 explains the categories and the codes.

**Table 2:** Codes corresponding to the analysis categories.

Analysis category	Code
SESI	0: does not include SESI 1: includes SESI
Co-creation	0: does not include co-creation 1: includes co-creation
Dimension 1: People who work for the company. Internal team.	0: does not include dimension 1 1: includes dimension 1
Dimension 2: People at whom the company’s social responsibility actions are aimed. Target population	0: does not include dimension 2 1: includes dimension 2
Dimension 3: People affected by the company’s main daily activity. General population.	0: does not include dimension 3 1: includes dimension 3

Please refer to Supplementary S1 File to download the Excel file.

### Quality assessment of the studies

The quality assessment of the studies includes the following steps, as specified in Table 3.

**Table 3:** Steps followed in the qualitative assessment and review instrument for the systematic review.

Step 1. Analysis of the Title and Abstract Screening	Initial screening of articles was based on their titles and abstracts. Articles were assessed for their relevance to the research questions and selection criteria. The inclusion criteria focused on articles that addressed the aspects of assessing a company’s social impact and analysing the actual social impact of a company. Two researchers carried out the initial evaluation following the predefined research objectives. Studies requiring a third assessment to determine their inclusion were subsequently reviewed by a third researcher.
Step 2. Full-Text Retrieval	Articles that met the inclusion criteria during the title and abstract screening phase had their full-text versions retrieved for further analysis. Studies that lacked the full text were excluded.
Step 3. Selection Criteria Assessment	The relevance of the studies was determined based on their alignment with the research questions and the defined selection criteria. This step ensured that only studies related to the research objectives were included.
Step 4. Dialogic Selection and Analysis Process	The study selection and analysis process were conducted through dialogue among the researchers involved. This dialogic approach aimed to enhance the reliability of the study selection process by minimising individual bias and ensuring consensus; this is dialogic reliability, where “dialogue is present throughout the process to guarantee the consistency of the analysis and characterised by being evidence-based in an egalitarian way that ensures the accuracy of the results’‘ [38]. This dialogic process is a fundamental characteristic of the development of the communicative methodology applied in the research according to Pulido et al [6].

Two analyses were undertaken concerning the initial part of the systematic literature review to address R1. The initial phase of the analysis involved selecting articles based on an abstract analysis derived from the defined queries. These queries centred around methodologies, measurements, or evaluation models for assessing companies’ social impact, either broadly or within a particular company, as detailed in Table 2. The total number of articles identified was 387, and the number o selected after reading the abstract was 81; 306 were excluded (see Table 4).

**Table 4:** Articles selected for further analysis.

Keywords	File	Results	Results selected
“social impact” AND companies	Query 01	288	55
“social impact” AND measurement AND companies	Query 02	27	10
“Environmental, Social, and Governance (ESG) measurement”	Query 03	72	16
“scientific evidence of social impact” AND companies	–	0	0
“scientifically validated evidence of improvement” AND companies	–	0	0
Total	–	387	81

**Fig 1:**
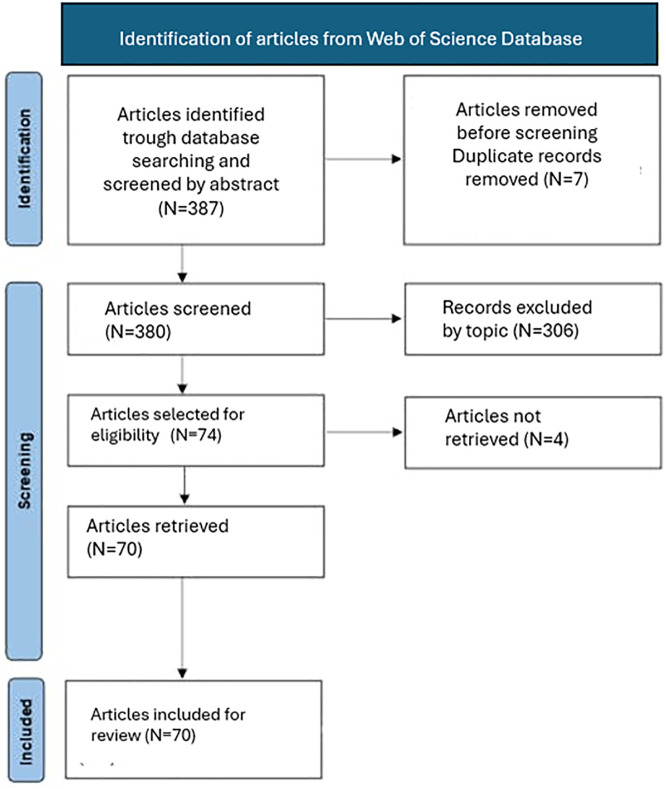
Flow diagram of systematic literature review.

A preliminary dataset-cleaning task was performed before completing the systematic literature review of the 81 found articles. A total of 387 records were identified and screened. During the initial screening phase, 7 duplicate records were removed, resulting in 380 unique articles screened by title and abstract. Based on relevance to the research topic, 306 records were excluded, and 74 articles were selected for full-text eligibility assessment. Of these, 4 articles were excluded due to lack of access, resulting in a final set of 70 articles included for further analysis (see Fig 1).

### Further analysis of the articles selected

A thematic analysis of the selected articles’ contributions was conducted to respond to the first research question: whether they included SESI or not and whether they were co-created with stakeholders or not. The social impact measured in the articles was classified according to the three dimensions of measurement and improvement defined in the Corporate Sustainability Reporting Directive, referred to in the introduction: a) Dimension 1: People who work for the company. Internal team. b) Dimension 2: People at whom the company’s social responsibility actions are aimed. Target population. c) Dimension 3: People affected by the company’s main daily activity. General population. These three dimensions were selected due to their connection with social impact, as well as because they reflect core areas highlighted by the European Commission’s frameworks, which encourage companies to evaluate their social responsibility efforts by considering impacts on both direct and indirect stakeholders to ensure a comprehensive, balanced, and transparent assessment. The CSRD proposal enhances the requirements for social and environmental reporting, including the assessment of corporate impacts on employees, communities, and society, moving towards a standardized approach to social impact reporting within the EU.

## Results

The results section is organised in two parts responding to the study’s research questions. The first section addresses the results obtained from the first research question (R1), specifically whether there are models or measurements for evaluating companies’ social impact based on SESI and co-creation. It is divided into three main subsections, corresponding to the three main themes selected for conducting the analysis. The second part addresses the second research question (R2) by presenting the pioneer model of the Dialogic Social Impact Analysis of Companies and Organisations (DSIACO), and it is presented in a single subsection.

### Absence of a Developed Model Combining SESI and Co-Creation for Evaluating Companies’ Social Impact

The analysis of the final list of seventy articles reveals that there is currently no sufficiently developed model that combines SESI and co-creation to evaluate companies’ social impact. Nonetheless, two main themes have emerged across the analysis of the seventy articles: a) Sixteen articles propose models that include initial evidence of SESI and/or, to some extent, co-creation, and b) Fifty-four articles propose models that do not gather SESI or co-creation. Table 5 summarises the two main themes into which the seventy articles analysed have been classified.

**Table 5:** The results of the further analysis have been developed.

Main Result	Number of articles
Assessment procedures with initial inclusion of SESI and/or co-creation	16 articles
Assessment Procedures without SESI and Co-Creation	54 articles

#### Assessment procedures with initial inclusion of SESI and/or co-creation.

Whereas the systematic literature review conducted did not identify any model that sufficiently developed for measuring the social impact of companies, sixteen articles were found to consider analysing evidence of specific social impact and/or the inclusion of stakeholders and end-users in what could be considered co-creation. Based on these two main themes, the authors have identified two groups of sub-themes. Table 6 summarises the number of articles assigned into each of the two sub-themes.

**Table 6:** Articles identified within the first main theme.

Sub-theme	Number of articles
Assessment procedures with initial inclusion of SESI	10
Assessment procedures with initial inclusion of SESI and some form of co-creation	6

*Assessment procedures considering SESI*. Ten articles were identified as primarily focusing on identifying indicators for evaluating companies’ social impact or gathering specific evidence of social improvements. For instance, Aranda et al. [39] highlight in their conclusions the utility and added value of quantifying and understanding how well the company is performing in terms of social performance. The authors point out that this enables the identification of strengths and weaknesses within the company and the prioritisation of actions towards achieving more socially responsible products in areas such as discrimination, health, and safety, among others. Furthermore, their conclusions also highlight the impact of this practice on the consumer’s perception. Cassano et al. [40] propose a framework for social and gender reporting in healthcare, stressing equal representation. Such a framework collects data on results in gender equality to assess the policies applied in the healthcare sector. Casanovas-Rubio et al. [41] introduce a deterministic decision-making method to quantify construction impacts on urban mobility. Their Mobility Impact Index supports multi-criteria evaluation in projects, with CSR (Corporate Social Responsibility) and sustainable impact implications. Furthermore, Rafiaani et al. [42] tackle the challenges of quantifying social indicators, considering various stakeholders and data availability. For instance, when considering workers, the indicator focuses on fair salary. For consumers, the indicators encompass end-of-life responsibility, company transparency, and consumer health and safety. Additionally, indicators for the local community include safe and healthy living conditions, secure living conditions, and local employment. In each case, specific evidence is gathered from the company and assigned a score based on a ranking system: 1 (indicating poor performance), 2 (denoting inadequate performance), 3 (reflecting satisfactory performance), or 4 (indicating excellent performance). On a different line, Hofflinger et al. [41]) studied large-scale forestry’s social impact. Their study reveals unexpected poverty and inequality effects, evaluating the social impact of the externalities of expanding the forestry industry in Indigenous and non-Indigenous populations located in six regions of Southern Chile in terms of employment, income poverty and income inequality. Other case studies were analysed for instance Do [43] delves into a social enterprise case, highlighting the simultaneous presence of social missions and financial profitability. In addition, Serreli et al. [44] assess the social and environmental impacts of microelectronics wastewater treatment. Similarly, Freelove and Gramatki [45] discuss the measuring of social value in infrastructure projects, utilising the Thames Tideway Tunnel as a case study. Moreover, Dulia et al. [46] evaluate the non-monetary effects of Advanced Air Mobility in Ohio, revealing positive stakeholder outcomes. Lastly, Caruso et al. [47] compare the social and environmental effects of mussel farming rafts in Spain, promoting innovative approaches for minimising impact.

*Assessment procedures considering SESI and co-creation*. The second group includes 6 articles that advance further by incorporating specific evidence of social impact and integrating the perspectives of end-users and/or other stakeholders, which could be categorised as a form of co-creation at some stage. For instance, Pasaribu and Vanclay [48] examine how to evaluate the activities of oil palm plantation companies in Indonesia in accordance with UNICEF’s Children’s Rights and Business Principles. They analyse the impact of these companies on children’s lives, their demonstration of respect for children’s rights, and possibilities for improvement. This research was collaboratively designed crafted and involved a diverse set of stakeholders, included children. Martinez and Komendantova [49] address the challenge of social opposition to renewable energy (RE) projects, particularly in the Global South. They evaluate the effectiveness of social impact assessments (SIAs) in Mexico’s RE sector, highlighting the constraints arising from institutional, regulatory, and implementation issues. In this case, the authors included different stakeholders’ voices in this evaluation. Moreover, Kuasoski et al. [50] identify and summarised the sustainable practices employed by red ceramic producers. The study accomplished this by examining the industry’s economic, environmental, and social impacts and shedding light on the complex interplay between production companies and local communities. This was achieved through a literature review and interviews with traditional producers. Osorio-Tejada et al. [51] apply social organisational life cycle assessment (SO-LCA) to evaluate the social performance of road transport supply chain companies across diverse contexts. They highlight the significance of labour rights and human rights in improving social performance, emphasising the influence of socio-economic development and cultural beliefs on social impact subcategories. The voices of stakeholders were collected for the recommendations stage. Moreovewer, Nath and Chaudhari [52] examine the influence of CSR initiatives by ITC Limited on stakeholders, with a specific focus on the learning outcomes of primary school students. Using data from rural areas in India, the authors reveal a substantial positive impact of corporate social responsibility (CSR) on stakeholders, particularly in enhancing learning outcomes. The latest contributions, highlighted by Morlà-Folch et al. [53], identify the Mondragon Cooperative (MC) as an example of Successful Cooperative Actions (SCAs) that can be transferred to other contexts. The study underscores the transferability of MC’s social impact to diverse corporate settings, illustrating the innovative application of qualitative research methodology for analysing and disseminating SCAs. This study employs the social impact approach, grounded in SESI and co-creation, utilising a communicative methodology that serves as a key point for further development.

Currently, companies use various methodologies and measurements to analyse their social impact. Nevertheless, many of these approaches found in the systematic literature review focus on only one, two, or three of the dimensions explained in the introduction and methodology sections.

#### Assessment procedures without SESI and Co-Creation.

Most of the articles identified in the systematic literature review do not include SESI. In addition, they do not involve co-creation with stakeholders and or end-users. The primary disparity lies in understanding “social impact” and how this concept is defined, which directly influences social impact evaluation. The fifty-four classified articles coded without SESI and Co-Creation can be divided into two groups of sub-themes: those analysing social impact from a specific perspective without co-creation and SESI (forty-one articles) and those attempting to propose evaluation methods within the ESG (Environmental, Social, and Governance) framework (thirteen articles). The two groups are presented in Table 7.

**Table 7:** Articles identified within the second main theme.

Sub-theme	Number of articles
Analysing social impact from a specific perspective without SESI and co-creation	41
Focusing specifically on ESG	13

*Analysing social impact from a specific perspective without SESI and co-creation*. The first group of articles (forty-one) is characterised by analysing the social impact from different specific cases. For instance, Brzustewicz et al. [54] focus on social impact, understood as actions that a company does outside its regular activities, such as corporate volunteering initiatives where companies collaborate with NGOs. Their analysis of four corporate volunteering projects reveal that social impact develops throughout the relationship, affecting individual, organisational, and societal levels. Similarly, Packt et al. [55] focused on evaluating social impact in product design, considering that the social impact from the initial stages of products can promote the social impact as an ex-ante methodology. Along the same lines, the role of the collaboration of stakeholders is highlighted by Rayment et al.[56] in the creation of impact based on the initial stage.

By contrast, Braig and Edinger-Schons [57] explore the Impact Measurement and Valuation (IMV) methods developed by companies, potentially becoming industry standards. They mainly examine the motivation and challenges of assuming this evaluation impact measurements, offering valuable insights for businesses implementing and evolving IMV. Likewise, Magomedova et al. [58] explored the impact of investment initiatives post-2008 crisis, focusing on social and environmental impact alongside financial returns, while Zebryte and Jorquera [59] analyse social entrepreneurship in Chilean Tourism B Corps, shedding light on how social entrepreneurs achieve impact-based business models, blending profit and social impact, with implications for traditional businesses. Finally, this article presents a Transparency Model for companies called TESUB, developed by Laguna et al. [60]. These authors argue that information transparency is crucial for evaluating the company’s social impact. Other articles analysed can be grouped by theme similarity: one theme is Corporate Social Responsibility Social Impact and Financial Impact Assessment [61–77]; another focused on social impact in cultural/sports companies or specific cultural contexts [56, 78–83]; and third addresses the influence of ICT and social media in the social perception of companies which does not itself constitute social impact [84–86].

*Focusing specifically on ESG*. The second group of articles (thirteen) within this theme shares a common objective: assessing the social impact while considering the ESG framework through various methodologies. For example, Widyawati [87] examines the quality of ESG ratings, revealing significantly distinct measurement criteria and concerns. Landi et al. [88] delve into the consequences of environmental, social, and governance ratings on corporate financial risk, investigating how investors perceive risk within sustainable strategies. Atkins et al. [89] explore the effectiveness of sustainability measurement metrics in the COVID-19 crisis, highlighting different ways companies responded to ESG measures during challenging times. Santamaria et al. [90] analyse the relationship between non-financial disclosure and ESG scores, specifically focusing on how the quality and quantity of disclosure impact ESG performance. Their research sheds light on the role of integrated reporting in promoting higher ESG scores and contributes to the effectiveness of disclosure measurements on ESG performance.

Another focus is the analysis of ESG rating agencies. Louche et al. [10] investigate how companies’ performance is assessed regarding decent work. Their study addresses the challenges rating agencies face in evaluating decent work and contribute to understanding how responsible investment and ESG ratings can promote and improve decent work within companies. An emerging field links the evaluation of ESG with psychological well-being, as Gärling et al. reflected [91]. These authors introduce the concept of sustainable investment’s potential role in promoting psychological well-being. Their article highlights the barriers and challenges of integrating ESG factors into investments and suggests that sustainable investment could help reduce material consumption levels, thus contributing to global sustainability.

Other articles explore assessment procedures through specific case studies. For instance, Hassani et al. [92] assessthe vulnerability of ESG factors among companies listed in the Dow 30. They analyse their societal and security impacts using the entropy weight method and suggest strategies to enhance governance communication. Yip et al. [93] examine ESG reporting in Hong Kong’s stock exchange. Their research spotlights the challenges small and medium-sized listed companies face in disclosing environmental key performance indicators (KPIs). Their findings further underscore the need for improved reporting quality and consistency among SMEs to enhance ESG data disclosure.

In addition to these cases, another topic of focus is global factors. For instance, Wang et al. [94] investigate the relationship between fintech development and corporate ESG performance in emerging markets, revealing that financially less constrained firms demonstrate stronger ESG performance in cities with more advanced fintech. In a related vein, Vieira De Castro et al. [95] adopt a holistic approach to sustainable commercial property business, aligning sustainability, ESG, and sustainable building practices to help companies internalise sustainability concepts across various operations. Moreover, Dempere and Abdalla [96] used a global dataset to examine the link between women’s empowerment and corporate ESG disclosure. Their focus is on how policies fostering gender diversity positively impact ESG disclosure, emphasising the importance of gender diversity in enhancing transparency, accountability, and corporate value. Another example is the study carried out by Sepulveda-Alzate et al. [97]. These authors explore the materiality of ESG information in Latin American companies using multivariate analysis to develop a materiality index. Their findings underscore the significance of issues like water management and climate change, thereby providing insights into the topics considered material by companies and stakeholders in various industries. Lastly, Sultana et al. [98] delve into how ESG considerations impact stock market investment decisions, particularly focusing on individual investors’ preferences and the role of investment purpose.

#### Dimensions included in the assessment procedure.

Finally, after analysing the seventy articles, it was determined that twenty of them [39, 40, 46, 49, 51–53, 55–60, 87–89, 92, 94, 95, 54] incorporated all three dimensions of analysis in their assessment procedure; Dimension 1: People who work for the company (internal teams) Dimension 2 – People at whom the company’s social responsibility actions are aimed – Target population; and Dimension 3, people who are affected the company’s main daily activity, general population. The inclusion of these three dimensions enhances understanding of how the social impact operates across these three dimensions, thereby providing a more comprehensive global overview of the social impact of the companies analysed. Table 8 identifies the number of articles that incorporate each dimension and all the dimensions.

**Table 8:** Summary of articles per dimension.

Dimension incorporated	Number of articles
Dimension 1. People who work for the company (internal team)	32
Dimension 2. People at whom the company’s social responsibility actions are aimed (target population)	47
Dimension 3. People who are affected the company’s main daily activity (general population)	46
All three dimensions	20

### Dialogic Social Impact Analysis of Companies and Organizations (DSIACO)

As far as the systematic literature review conducted, the authors did not identify any model sufficiently developed for evaluating the social impact of companies that integrates SESI and co-creation as valued dimensions. Therefore, Dialogic Social Impact Analysis of Companies and Organizations (DSIACO) represents a step forward in advancing this field, as it is a pioneer model for evaluating the social impact of companies and organisations based on the dimensions previously discussed. This model has emerged from the analysis of the social impact of multiple organisations carried out by CREA [1, 20–30] since its inception in 1991. The scientific community and companies could both use the DSIACO model to evaluate social impact. The scientific community, because such evaluation t is a requirement of all sciences, wheter funded or not, as the evaluation of sciences also depends on the social impact of their contributions to society. Likewise, companies and organisations must report on their social impact. For instance, 11,700 European Union companies are obliged to report on the social impact and need models based on SESI. Some companies, such as pharmaceuticals, are already applying science-based issues that need an assessment model based on SESI.

The DSIACO model is dialogic because it is based on contributions of *Dialogic Communicative Acts* [99] and the contributions of the role of the dialogue in companies as defined in the Dialogic Society [4]. For a communicative act to be dialogical [99], it must meet two conditions: it must be based on illocutionary communicative acts (thus, based on sincerity and the pursuit of consensus), and dialogical interactions must predominate (based on dialogue without coercion). Conversely, power communicative acts encompass perlocutionary communicative acts (aimed at achieving an action) and the predominance of power interactions arising from the speaker’s own intentionality and/or social structural inequalities. DSIACO is based on the contributions regarding dialogue in companies as outlined in The Dialogic Society [4] defined in this paragraph:

Even most of those who agree with the statement that organisations are having a dialogic transformation deny this process in the case of enterprises due to the fact that their main goal is to maximise profit. Nevertheless, current scientific analysis detects more dialogic processes in many companies than ever before; instead of reducing their economic success, many of them are increasing it thanks to this dialogic transformation. Of course, this is not the case for all of them, the result depends on the quality of their dialogic orientation. For that reason, it is very important for science and society to analyse the companies that are making clear steps at the same time towards dialogue and towards economic success [4].

Thus, considering all the previous contributions, the dialogue in DSIACO plays a crucial role. The process of collecting data on companies’ social impact should be dialogic in the terms defined in dialogic communicative acts, and it is based on the statement that companies that address the dialogic process towards economic and social impact have more success. Consisting with this view, Morlà-Folch et al. [53] contributions bring us closer to the objective set by pioneering the Successful Cooperative Actions (SCAs), based on the case study of the Mondragon Corporation. The study identifies the key actions of this business model that can be transferred to other corporate contexts. In line with this, the CREA network of researchers [20] has led the study of the social impact of scientific research in all areas of knowledge. In this way, the basis for the study and measurement of the social improvements developed by research has been established. However, the analysis of social impact has not always been the priority for either research or companies. In the European context, the Directorate-General for Research of the European Commission (EC), through the document “Monitoring the Impact of EU Framework Programmes: Expert Report” [100], marked a tipping point in the debate on social impact. This document established guidelines to identify and measure the scientific, political, and especially social impact of EC-funded research across all knowledge areas, aiming to develop a permanent system for selecting, tracking, and evaluating research projects’ results.

One of the first distinctions developed by Flecha under his leadership of the Expert Group Chair on Evaluation Methodologies for the Interim and Ex-post evaluations of Horizon 2020, DG Research and Innovation (European Commission), composed of 17 members of all scientific disciplines, was precisely the definition and clarification of the three types of impact that can arise from research. Scientific impact refers to the production and publication (articles, book chapters, etc.) of research results. It encompasses the impact factor (and its derivatives, i.e., AL-METRICS, H-index), the presentation of such research results at conferences or other academic forums, and the opportunities the research results create for conducting subsequent research. A distinction is made between academic impact, understood as the intellectual contribution a researcher makes to academia, and socioeconomic impact, which extends beyond academia [101].

Addressing social impact remains a current challenge worldwide. The Literature highlights a variety of processes that could be defined as improvements or social impact. For instance, within Australia’s Research Quality Framework (RQF), research impact definitions generated by the scientific community included ‘increasing the nation’s social capital, economy, and natural and cultural heritage’ [102]. Other approaches to conceptualising social impact are similar to those used for economic impact. For example, the U.S. STARMETRICS initiative by Weinberg et al. [103] identified the volume of job creation resulting from research. Flecha [104] distinguishes between scientific impact, dissemination, political impact, and social impact and argues that social impact can be understood as the culmination of these three stages of research. Therefore, the social impact of research occurs when the published and disseminated results, which have been transferred to a policy or social initiative, produce improvements in society’s declared goals.

In this context, following several years of diligent work, the European Commission’s research has reached a consensus, which has been scientifically validated, regarding all components of social impact. The data sources, including quantitative and qualitative information, must be made explicit within these components. Crucially, the individuals providing this data — the citizens, consumers, or stakeholders — play an instrumental role in this evaluation. Thus, the methodologies for this kind of evaluation should consider the perspectives of citizens and consumers and should be cost-effective. To this end, a newly developed methodology [23], Social Media Analytics, specifically Social Impact in Social Media (SISM) methodology, has been used. This method enables the collection of evaluations, opinions, and data from citizens who are increasingly active on various social networks.

The validity of the methodologies is enhanced through the the adoption of a co-creation approach. This involves engaging with various entities, including academic, corporate, and social organisations. For example, CREA ‘s dialogue included Nobel Prize laureates from diverse scientific fields, citizens, consumers, and corporations worldwide. This co-creation process ensures that the findings reflect a broad consensus. An illustrative example of this co-creation process involves Nobel Prize laureate Harald zur Hausen. Concerned about differing national responses to his discovery, zur Hausen was interested in understanding which approach had better outcomes: mass distribution of the papilloma vaccine or annual health check-ups. By utilising social media analytics, we could gather real-time information about the consequences of each approach. This method proved cost-effective and captured the evolving situation more efficiently than traditional surveys.

At present, there are three main approaches to the analysis of social impact measurement that different agencies are currently utilising. The most common approach involves good practices of transference measurement. A more sophisticated method involves good practices of social impact measurement, which focuses not just on transferring the knowledge but also on the consequences of each approach. Lastly, the most advanced approach involves successful practices of social impact measurement, which includes a scientific evaluation of the effectiveness of various actions and their potential for application in other contexts. These methodologies have been tested with companies like Orbea, a member of the Mondragon Group. The findings reveal that good practices of social impact measurement involve analysing the consequences of a company’s actions on all citizens. Various metrics, such as the Gini index, can be used to gauge social impact. Examining successful practices of measurement of social impact helps to explore why the selected actions have a successful social impact.

In light of the previous the previous contribution, the pioneer contribution of the Dialogic Social Impact Analysis of Companies and Organizations (DSIACO) [19] is the analysis of the social impact that one company or organisation already has and the actions that can be implemented to improve it. This analysis model has the following characteristics: a) it is based on SESI, overcoming common errors such as confusing dissemination or transference with social impact. b) it applies interdisciplinary and global methodologies since these are the best to clarify the current social impact that the company or organisation is having even without being aware. c) It focuses on successful actions of social impact (SASI), that have been demonstrated to improve social impact with fewer resources.

The DSIACO model analyses companies’ social impact through a thorough study of their compliance with the Sustainable Development Goals, collecting those relevant to the company’s field of work, as well as the global objectives chosen by citizens. Once the objectives have been selected, the related social indicators are developed, considering the following items: 1. Indicator, 2. Definition, 3. Rationale, 4. Sources, 5. Who is providing the information, 6. Methodology for indicator computation, 7. Frequency of measurement, 8. Estimated cost of data collection, 9. Level of reporting burden.

To determine the company’s improvement, the quantitative indicators must be comparable with those already existing in official reports or those of surrounding organisations. When analysing the indicators, a thorough analysis must be made to avoid superficial analyses that may overestimate the company’s impact. For example, if the number of jobs created is analysed, the number of jobs destroyed in other companies in the surrounding area must be deducted.

**Fig 2:**
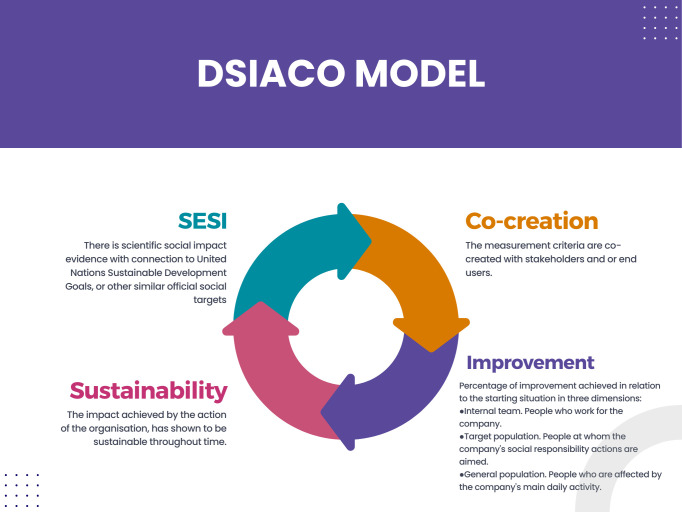
DSIACO model [19].

In the DSIACO model (see Figure 2), Social Impact Scores are assigned according to the evidence of social improvements associated with official social targets (such as the UN Sustainable Development Goals, the Europe 2020 agenda, and others) and the evidence related to the following criteria:

- There is scientific social impact evidence with a connection to United Nations Sustainable Development Goals or other similar official social targets.
- The measurement criteria are co-created with stakeholders and or end users.
- Percentage of improvement achieved to the starting situation in three dimensions:
  - Internal team. People who work for the company.
  - Target population. People at whom the company’s social responsibility actions are aimed.
  - General population. People who are affected by the company’s main daily activity.
- Sustainability. The impact achieved by the action of the organisation, has shown to be sustainable throughout time.

In the following Table 9 (developed by authors), scores range from 1 to 10, according to the degree of fulfilment of the previous five criteria:

**Table 9:** Criteria and score for the assessment of social impact of companies and organizations.

Criteria	Score
1) Connection to United Nations Sustainable Development Goals or other similar official social targets.	
2) Achievement of an improvement towards the internal team.	
3) Achievement of an improvement towards the target population.	
4) Achievement of an improvement towards the general population.	
5) Sustainability of the social impact.	
6) Publication in scientific journals	
7) Publication by Policy documents	
8) Engagement in co-creation	
The impact meets ALL the criteria.	10
The impact meets criteria 1–3 and 4 of the other 5 criteria.	9
The impact meets criteria 1–3 and 3 of the other 5 criteria.	8
The impact meets criteria 1–3, and 2 of the other 6 criteria.	7
The impact meets criteria 1–3, and 1 of the other 6 criteria.	6
The impact meets criteria 1 and 2, and 4 of the other 6 criteria.	5
The impact meets criteria 1 or 2, and 3 of the other 6 criteria.	4
The impact meets criteria 1 or 2, and 2 of the other 6 criteria.	3
The impact meets criteria 1 or 2, and 1 of the other 6 criteria.	2
The impact meets criteria 1 or 2.	1

For organisations with more than one identified social impact, a score will be assigned for each. The final score for a given organisation’s social impact will correspond to be the highest score obtained. A score of 1 is already a good result, as it indicates that there is some measurable social impact.

In conclusion, understanding social impact requires a comprehensive analysis that encompasses transfer of knowledge, social impact on health and well-being, and the overall societal consequences of a company’s operations. Ensuring that our methods for measuring social impact are both valid and inclusive, and they represent a broad consensus is fundamental for ensuring the excellent assessment of social impact. With the help of diverse methodologies, one of them, Social Impact in Social Media (SISM) methodology [23], and the inclusion of diverse voices in the conversation, we can better analyse and improve social impact.

## Discussion

This study presents, for the first time in a scientific article, a developed model for evaluating the social impact of companies and organisations based on SESI and Cocreation, thereby addressing a significant gap in existing research. It responds to the increasing demand for methods to measure social impact within the corporate context. While companies inherently aim for economic and social goals, there is a lack of common parameters to measure the social impact from a scientific perspective. This study identifies this gap and presents DSIACO (Dialogic Social Impact Analysis of Companies and Organizations), based on the major contributions of social impact assessment based on the SESI [2] perspective. Moreover, the DSIACO model [19] incorporates a dialogic approach to social impact assessment based on scientific evidence. This evidence demostrates that integrating dialogue into assessment procedures significantly enhances the outcomes, decision-making processes, and stakeholder involvement in company evaluations. This model contributes to the extensive dialogue on dialogic evaluation [105], which has a long history of reflecting on how dialogue can improve assessment procedures.

Before presenting the DSIACO model, a systematic literature review was conducted to identify any existing models based on SESI and co-creation. This study confirms the absence of a comprehensive model for evaluating companies’ social impact in the systematic literature review conducted, which integrates SESI and co-creation as an added dimension, even though it is not required for achieving social impact in the selected and analysed dataset. The analysis reveals that none of the identified models adequately combine these two dimensions to assess social impact effectively. The articles analysed are divided into distinct categories. The first group (forty-one articles) examines social impact from specific perspectives without incorporating SESI or co-creation. A second group (thirteen articles] focuses on assessing social impact within the ESG framework, considering environmental, social, and governance factors. These articles explore the relationships between non-financial disclosure and ESG scores, as well as the implications of ESG ratings on corporate risk and sustainability metrics during the COVID-19 pandemic. Other articles investigate how ESG rating agencies assess decent work and examine the link between fintech development and corporate ESG performance in emerging markets. A third group (sixteen articles) involves the inclusion of initial SESI in assessment procedures. Some articles identify indicators for evaluating social impact, while others concentrate on specific evidence of social improvements. Examples include assessing the impact of oil palm plantation companies on children’s rights, evaluating social and gender reporting in healthcare, and quantifying construction impacts on urban mobility. Within these groups, certain articles (ten) consider initial evidence of specific social impact and highlight indicators for evaluating social performance. The second subgroup of articles (six) presents an advancement by including end-user and stakeholder voices. A group of twenty articles included in their assessment procedures three dimensions: the internal team, the target population of the company’s social responsibility actions, and the general population affected by the company’s main daily activities. Incorporating these dimensions offers a comprehensive overview of the social impact of the companies analysed.

Despite the exhaustive analysis, no article to date has presented a model sufficiently developed to measure companies’ social impact based on SESI. Consequently, through the review of the scientific literature and previous work on social impact analysis, this article makes a pioneering contribution to the Dialogic Social Impact Analysis of Companies and Organisations (DSIACO). DSIACO refers to the analysis of the social impact that a company or organisation is already having and the actions it can implement to improve such social impact based on a scientific perspective. As mentioned in the previous section, the Social Impact Scores in DSIACO model are assigned according to the evidence of social improvements associated with official social targets (such as the UN Sustainable Development Goals, the Europe 2020 agenda, and others) and the evidence related to the following criteria: 1) Scientific social impact evidence with connection to the United Nations’ Sustainable Development Goals, or other similar official social targets, 2) The measurement criteria are co-created with stakeholders and or end-users, 3) The percentage of improvement achieved in relation to the starting situation in three dimensions: ‘internal team’ (people who work for the company), ‘target population’ (people at whom the company’s social responsibility actions are aimed) and ‘general population’ (people who are affected by the company’s main daily activity), and 4) Sustainability, which refers to whether the impact achieved by the action of the organisation has shown to be sustainable throughout time. The DSIACO model contributes to addressing the gap identified in the systematic literature review of assessment procedures for companies’ social impact, based on SESI scientific. The model will also be enriched by the experience gained in the first pilots of applying the proposed methodology.

While this study makes a meaningful contribution to the literature, several limitations must be acknowledged. First, the systematic review was limited to articles indexed in Web of Science, which, although rigorous, may exclude relevant studies published in other databases or grey literature. Second, while the DSIACO model is presented as a theoretically grounded and innovative contribution, its empirical validation is ongoing and not the focus of this paper. As such, the practical application and effectiveness of DSIACO across diverse contexts remain to be thoroughly tested.

With the regard to the future research directions, several paths should be pursued. First, a comparative analysis between DSIACO and existing models identified in this review could offer valuable insights into its relative strengths and areas for refinement. Second, empirical studies applying DSIACO in different sectors and regions are needed to assess its adaptability, scalability, and real-world impact. Additionally, further research could explore how DSIACO can be integrated into regulatory frameworks, such as the Corporate Sustainability Reporting Directive (CSRD) or ESG reporting, potentially strengthening the connection between corporate reporting practices and scientifically validated social impact.

## Conclusion

The DSIACO model aims to be a tool that serves scientists, politicians, and above all, companies and citizens in accounting for the social improvements produced by companies and organisations, as well as the further improvements that such entities can develop to deepen their impact on the social level.

Moreover, the DSIACO model contributes to the existing knowledge in several significant ways: a) The DSIACO model enhances a more thorough and evidence-based approach to understanding the social impact of companies and organisations on society. This focus on scientific evidence of social impact strengthens the credibility and reliability of social impact assessments; b) Co-Creation and Dialogue: The DSIACO model introduces the concept of co-creation, emphasising the involvement of stakeholders and end-users in the measurement criteria for social impact. This dialogic approach ensures that diverse perspectives are considered, leading to more comprehensive and inclusive assessments; c) The DSIACO model provides a comprehensive framework for evaluating social impact, encompassing dimensions such as the impact on the internal team, target population, and general population and d) The DSIACO model encourages organisations to publish their findings in scientific journals, contributing to the body of knowledge on social impact assessment. In summary, the DSIACO model advances the field of social impact assessment by promoting scientific evidence of social impact and a dialogic and comprehensive approach to evaluating the impact of companies and organisations on society.

## Supporting information

S1 File Tables 1–8. (DOCX)

S2 File Flow diagram of systematic literature review. (TIF)

S3 File Criteria and score for the assessment of social impact of companies and organizations. (DOCX)

S4 File Figure 2 DSIACO model. (TIFF)

S5 File Supporting information- systematic literature review dataset. (XLSX)

S6 File Supporting information-prisma 2009 checklist. (PDF)
