# Correction: Dialogic Social Impact Analysis of Companies and Organizations (DSIACO): A pioneer model for evaluating social impact of companies and organizations

**DOI:** 10.1371/journal.pone.0351979

**Published:** 2026-06-16

**Authors:** Mar Joanpere, Ana Burgues-Freitas, Cristina Pulido, Adriana Aubert, Elisabeth Torras-Gómez, Maria Vieites, Marta Soler-Gallart, Ramon Flecha

The Competing interests statement for this article is incorrect. The correct Competing interests statement is as follows: Some authors of this publication (Ramón Flecha, Marta Soler-Gallart, María Vieites, and Elisabeth Torras-Gómez) served on the governing board of Social Impact SL during the study and publication period. Ramón Flecha served as President of the Board and María Vieites served as Administrator. María Vieites was also employed by Social Impact SL. The remaining board members did not receive profit distributions, financial compensation, or personal financial benefit from the company. The other co-authors (Mar Joanpere, Adriana Aubert, Ana Burgués and Cristina Pulido) also participated as collaborators in one report developed within Social Impact SL. Their participation took place during their personal time and without remuneration, profit distribution, financial compensation, or personal financial benefit.
