# Supplementary material for: Dialogic Social Impact Analysis of Companies and Organizations (DSIACO): A pioneer model for evaluating social impact of companies and organizations
Source: PLoS One. 2025 Oct 27;20(10):e0334833. doi: 10.1371/journal.pone.0334833 (PMC12558549; doi:10.1371/journal.pone.0334833)
Supplement: S1 File — (DOCX) [file pone.0334833.s001.docx]

Table 1. Keyword combinations used in queries conducted in Web of Science

| **Keyword combinations** | **Query number** |
| --- | --- |
| “social impact” AND companies | Query 01 |
| “social impact” AND measurement AND companies | Query 02 |
| “Environmental, Social, and Governance (ESG) measurement” | Query 03 |
| “scientific evidence of social impact” AND companies | - |
| “scientifically validated evidence of improvement” AND companies | - |

Table 2. Codes corresponding to the analysis categories

| **Analysis category** | **Code** |
| --- | --- |
| SESI | 0: does not include SESI  1: includes SESI |
| Co-creation | 0: does not include co-creation  1: includes co-creation |
| Dimension 1: People who work for the company. Internal team. | 0: does not include dimension 1  1: includes dimension 1 |
| Dimension 2: People at whom the company's social responsibility actions are aimed. Target population | 0: does not include dimension 2  1: includes dimension 2 |
| Dimension 3: People affected by the company's main daily activity. General population. | 0: does not include dimension 3  1: includes dimension 3 |

Table 3. Steps followed in the Qualitative Assessment and Review Instrument for the Systematic Review

| Step 1.  Analysis of the Title and Abstract Screening | Initial screening of articles was based on their titles and abstracts. Articles were assessed for their relevance to the research questions and selection criteria. The inclusion criteria focused on articles that addressed the aspects of assessing a company's social impact and analysing the actual social impact of a company.  Two researchers carried out the initial evaluation following the predefined research objectives. Studies requiring a third assessment to determine their inclusion were subsequently reviewed by a third researcher. |
| --- | --- |
| Step 2. Full-Text Retrieval | Articles that met the inclusion criteria during the title and abstract screening phase had their full-text versions retrieved for further analysis. Studies that lacked the full text were excluded. |
| Step 3. Selection Criteria Assessment | The relevance of the studies was determined based on their alignment with the research questions and the defined selection criteria. This step ensured that only studies related to the research objectives were included. |
| Step 4. Dialogic Selection and Analysis Process | The study selection and analysis process were conducted through dialogue among the researchers involved. This dialogic approach aimed to enhance the reliability of the study selection process by minimising individual bias and ensuring consensus; this is dialogic reliability, where “dialogue is present throughout the process to guarantee the consistency of the analysis and characterised by being evidence-based in an egalitarian way that ensures the accuracy of the results'' [[40]](https://paperpile.com/c/4OIlFI/bu7Wq/?locator=384). This dialogic process is a fundamental characteristic of the development of the communicative methodology applied in the research according to Pulido et al [6]. |

Table 4. Articles selected for further analysis

| **Keywords** | **File** | **Results** | **Results selected** |
| --- | --- | --- | --- |
| “social impact” AND companies | Query 01 | 288 | 55 |
| “social impact” AND measurement AND companies | Query 02 | 27 | 10 |
| “Environmental, Social, and Governance (ESG) measurement” | Query 03 | 72 | 16 |
| “scientific evidence of social impact” AND companies | - | 0 | 0 |
| “scientifically validated evidence of improvement” AND companies | - | 0 | 0 |
| Total | - | 387 | 81 |

Table 5.  The results of the further analysis have been developed.

| **Main Result** | **Number of articles** |
| --- | --- |
| Assessment procedures with initial inclusion of SESI and/or co-creation | 16 articles |
| Assessment Procedures without SESI and Co-Creation | 54 articles |

Table 6. Articles identified within the first main theme.

| **Sub-theme** | **Number of articles** |
| --- | --- |
| Assessment procedures with initial inclusion of SESI | 10 |
| Assessment procedures with initial inclusion of SESI and some form of co-creation | 6 |

Table 7. Articles identified within the second main theme.

| **Sub-theme** | **Number of articles** |
| --- | --- |
| Analysing social impact from a specific perspective without SESI and co-creation | 41 |
| Focusing specifically on ESG | 13 |

Table 8. Summary of articles per dimension.

| **Dimension incorporated** | **Number of articles** |
| --- | --- |
| Dimension 1. People who work for the company (internal team) | 32 |
| Dimension 2. People at whom the company’s social responsibility actions are aimed (target population) | 47 |
| Dimension 3. People who are affected the company’s main daily activity (general population) | 46 |
| All three dimensions | 20 |
