# Supplementary figures and images for: Dialogic Social Impact Analysis of Companies and Organizations (DSIACO): A pioneer model for evaluating social impact of companies and organizations
Source: PLoS One. 2025 Oct 27;20(10):e0334833. doi: 10.1371/journal.pone.0334833 (PMC12558549; doi:10.1371/journal.pone.0334833)

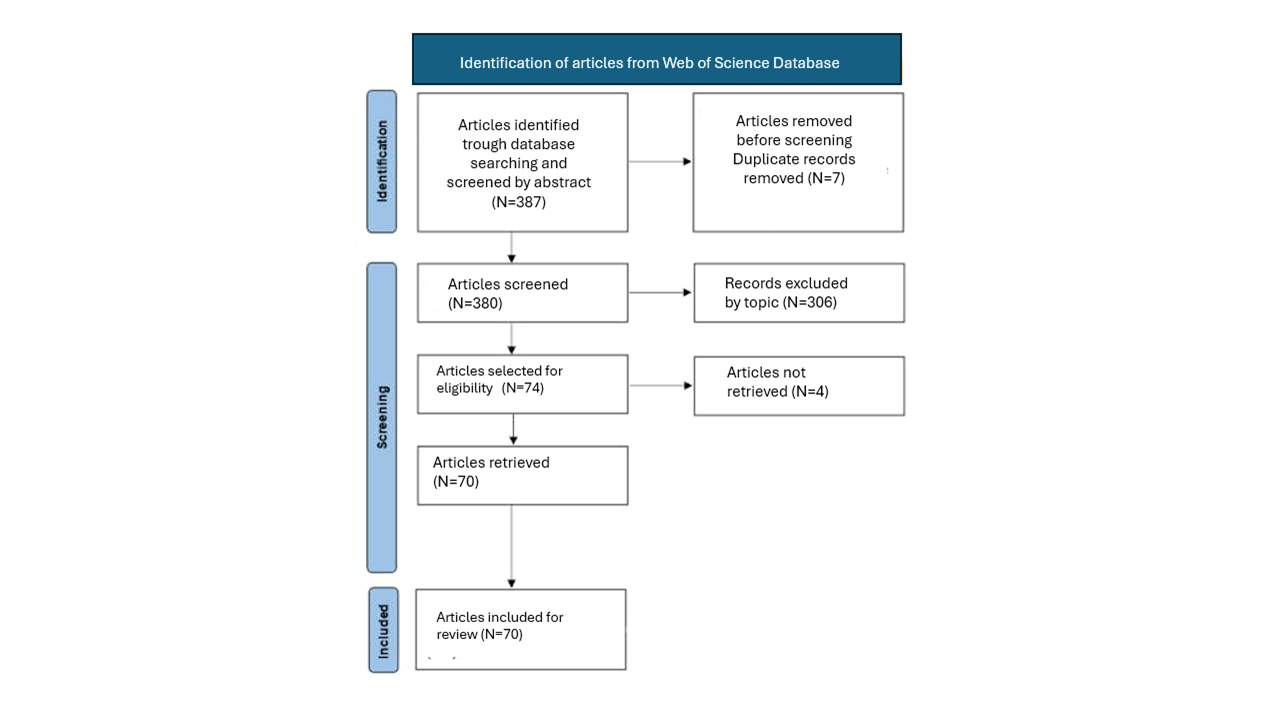

Supplement: S2 File — (TIF) [file pone.0334833.s002.tif]

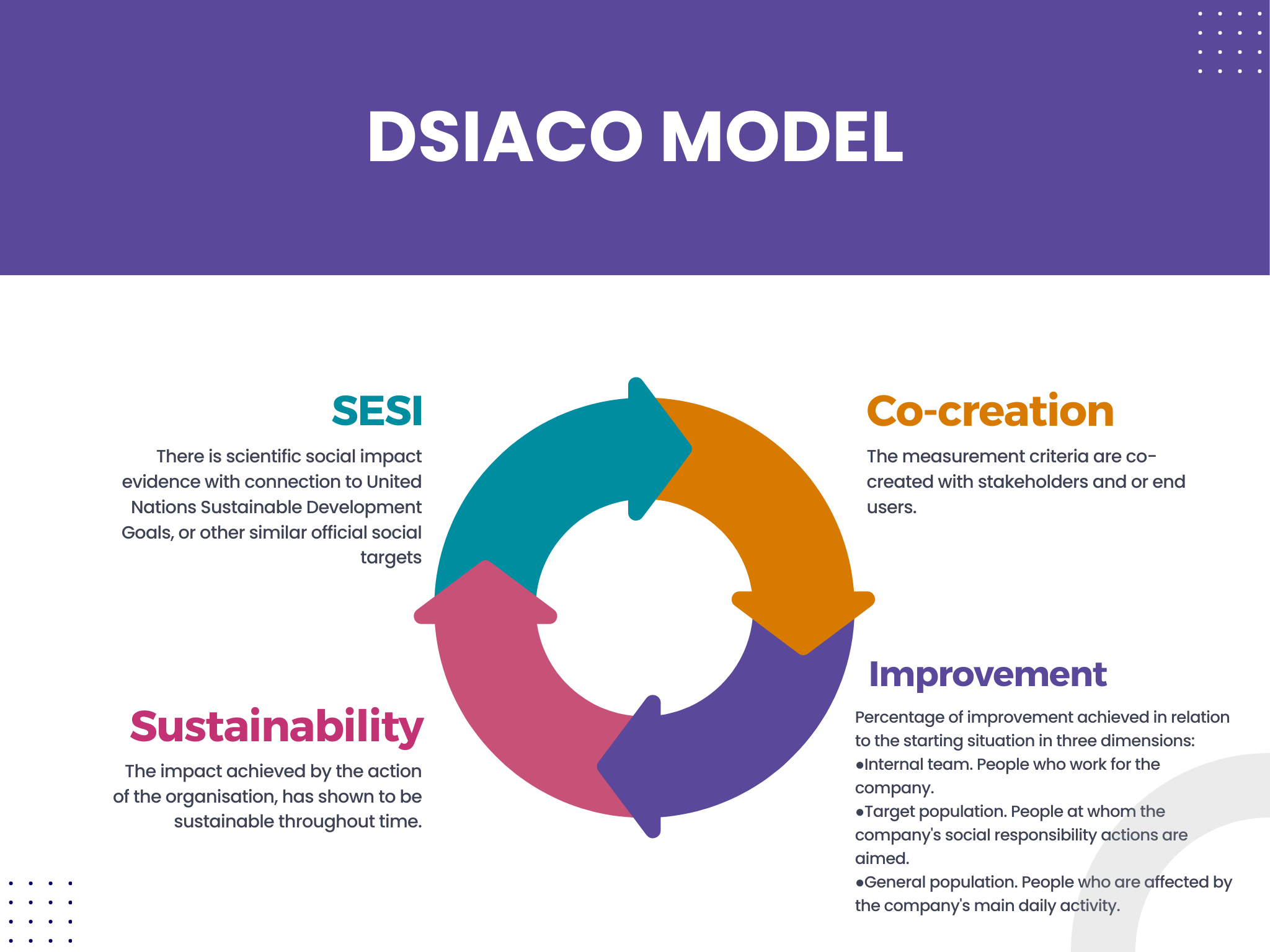

Supplement: S4 File — (TIFF) [file pone.0334833.s004.tiff]
