# Supplementary material for: Dialogic Social Impact Analysis of Companies and Organizations (DSIACO): A pioneer model for evaluating social impact of companies and organizations
Source: PLoS One. 2025 Oct 27;20(10):e0334833. doi: 10.1371/journal.pone.0334833 (PMC12558549; doi:10.1371/journal.pone.0334833)
Supplement: S3 File — (DOCX) [file pone.0334833.s003.docx]

Table 9. Criteria and Score for the Assessment of Social Impact of Companies and Organizations

| **Criteria** | **Score** |
| --- | --- |
| 1) Connection to United Nations Sustainable Development Goals or other similar official social targets.  2) Achievement of an improvement towards the internal team.  3) Achievement of an improvement towards the target population.  4) Achievement of an improvement towards the general population.  5) Sustainability of the social impact.  6) Publication in scientific journals  7) Publication by Policy documents  8) Engagement in co-creation |  |
| The impact meets ALL the criteria. | 10 |
| The impact meets criteria 1 to 3 and 4 of the other 5 criteria. | 9 |
| The impact meets criteria 1 to 3 and 3 of the other 5 criteria. | 8 |
| The impact meets criteria 1 to 3, and 2 of the other 6 criteria. | 7 |
| The impact meets criteria 1 to 3, and 1 of the other 6 criteria. | 6 |
| The impact meets criteria 1 and 2, and 4 of the other 6 criteria. | 5 |
| The impact meets criteria 1 or 2, and 3 of the other 6 criteria. | 4 |
| The impact meets criteria 1 or 2, and 2 of the other 6 criteria. | 3 |
| The impact meets criteria 1 or 2, and 1 of the other 6 criteria. | 2 |
| The impact meets criteria 1 or 2. | 1 |
